# Ezh2 Regulates Activation-Induced CD8^+^ T Cell Cycle Progression *via* Repressing *Cdkn2a* and *Cdkn1c* Expression

**DOI:** 10.3389/fimmu.2018.00549

**Published:** 2018-03-26

**Authors:** Guobing Chen, Kalpana Subedi, Sayantan Chakraborty, Alexie Sharov, Jian Lu, Jaekwan Kim, Xiaofan Mi, Robert Wersto, Myong-Hee Sung, Nan-ping Weng

**Affiliations:** ^1^Lymphocyte Differentiation Section, Laboratory of Molecular Biology and Immunology, National Institute on Aging (NIH), Baltimore, MD, United States; ^2^Transcription Systems Dynamics and Biology Unit, Laboratory of Molecular Biology and Immunology, National Institute on Aging (NIH), Baltimore, MD, United States; ^3^Laboratory of Genetics and Genomics, National Institute on Aging (NIH), Baltimore, MD, United States; ^4^Flow Cytometry Unit, National Institute on Aging (NIH), Baltimore, MD, United States

**Keywords:** EZH2, CD8^+^ T cells, cell cycle, CDKN2A, CDKN1C

## Abstract

Transition from resting to cell cycle in response to antigenic stimulation is an essential step for naïve CD8^+^ T cells to differentiate to effector and memory cells. Leaving the resting state requires dramatic changes of chromatin status in the key cell cycle inhibitors but the details of these concerted events are not fully elucidated. Here, we showed that Ezh2, an enzymatic component of polycomb repressive complex 2 (PRC2) catalyzing the trimethylation of lysine 27 on histone 3 (H3K27me3), regulates activation induced naïve CD8^+^ T cells proliferation and apoptosis. Upon deletion of Ezh2 during thymocyte development (Ezh2^fl/fl^Cd4Cre^+^ mice), naive CD8^+^ T cells displayed impaired proliferation and increased apoptosis in response to antigen stimulation. However, naive CD8^+^ T cells only had impaired proliferation but no increase in apoptosis when Ezh2 was deleted after activation (Ezh2^fl/fl^GzmBCre^+^ mice), suggesting cell cycle and apoptosis are temporally separable events controlled by Ezh2. We then showed that deletion of Ezh2 resulted in the increase in expression of cyclin-dependent kinase inhibitors Cdkn2a (p16 and Arf) and Cdkn1c (p57) in activated naïve CD8^+^ T cells as the consequence of reduced levels of H3K27me3 at these two gene loci. Finally, with real time imaging, we observed prolonged cell division times of naïve CD8^+^ T cells in the absence of Ezh2 post *in vitro* stimulation. Together, these findings reveal that repression of *Cdkn1c* and *Cdkn2a* by Ezh2 plays a critical role in execution of activation-induced CD8^+^ T cell proliferation.

CD8^+^ T cells deliver the main antigen-specific cytotoxic function in immune response. The typical sequential events of a CD8^+^ T cell response include proliferation of antigen-specific naïve CD8^+^ T cells post-antigenic challenge to generate sufficient number of effector cells, eliminate antigen-expressing target cells, and differentiate into long-lived memory CD8^+^ T cells (1, 2). At each stage of differentiation, CD8^+^ T cells gain different functions from cytokine production to cytotoxicity in effector cells and properties of rapid responsibility and longevity in memory cells (3–5). The foundation of differentiation is the ability of activation-induced cell cycle progression, which requires precise chromatin modifications in a timely fashion at specific gene loci through concerted efforts of multiple chromatin modifiers (6, 7). How these sequential events are coordinated at the chromatin level in CD8^+^ T cells during activation have just begun to be understood.

The cell division cycle is directly controlled by both positive regulators (cyclins and cyclin-dependent kinase, Cdks) and negative regulators (cyclin-dependent kinase inhibitors, Cdkn). Two families of Cdkns are active in resting T cells. The inhibitors of CDK4 family, including Cdkn2a [p16 and alternative reading frames (Arf)], Cdkn2b (p15), Cdkn2c (p18), and Cdkn2d (p19), and the Cip/Kip family, including Cdkn1a (p21), Cdkn1b (p27), and Cdkn1c (p57), play an essential role in maintaining the G0/G1 phase during T cell development, differentiation, and function (8, 9). Studies of individual Cdk inhibitor using genetically modified mice model reveal their distinct roles in T cell development and differentiation. Cdkn2a-deficient mice have thymic hyperplasia and increased peripheral T cell proliferation whereas increased expression of Cdkn2a leads to arrest of thymocyte development at DN3 stage (10, 11). However, deletion of the alternative reading frames (Arf) of Cdkn2a in mice does not affect cell proliferation but enhance apoptosis of dividing cells (12). Cdkn1c regulates cell division and is also involved in the p53-dependent apoptosis pathway (13, 14). Less is known about the upstream events of the epigenetic regulation of these cell cycle regulators during CD8^+^ T cell activation and proliferation.

Ezh2 is a methyltransferase that catalyzes trimethylation of Lys-27 on H3 (H3K27me3) and is a core component of the polycomb repressive complex-2 (15–17). Ezh2 is involved in important development processes such as X chromosome imprinting, early embryonic development, and multipotent progenitor’s fate (15–17). Ezh2 plays critical roles in T cell differentiation and function (18, 19). Ezh2 was first reported to interact with Vav1 of TCR signaling *via* actin polymerization-dependent processes (20). Ezh2 is also capable of positively regulating cytokine expression during CD4^+^ T cell differentiation (21–23) and has been implicated in Treg cell differentiation through repressing corresponding transcription factors (24, 25). Another important phenotype of Ezh2-deficient T cells is enhanced T cells apoptosis during immune response (26, 27). More recently, it has been demonstrated that Ezh2 maintains the fate of terminal effector CD8^+^ T cells by repressing the pro-memory gene sets (28). Although it is reported that Ezh2 regulates *Cdkn2a* and *Cdkn1c* loci in tumor cell lines (29, 30), the involvement of Ezh2 in activation induced CD8^+^ T cell cycle progression and apoptosis has not been fully characterized.

Here, we focused on the cell cycle progression and apoptotic events during naïve CD8^+^ T cell activation using two T cell-specific Ezh2 knockout models (Cd4Cre as a stable deletion and GzmbCre as an activation-induced deletion) and direct monitoring of naïve CD8^+^ T cell divisions using long-term live imaging. We found that stable deletion of Ezh2 (Ezh2^fl/fl^Cd4Cre^+^) had both impaired proliferation and enhanced apoptosis whereas activation-induced deletion of Ezh2 (Ezh2^fl/fl^GzmbCre^+^) was only impaired in proliferation but not apoptosis. At the gene level, Ezh2 repressed *Cdkn2a* (p16 and Arf) and *Cdkn1c* (p57), both of which are essential for naive CD8^+^ T cells entering cell cycle post-activation. Furthermore, in the absence of Ezh2, naive CD8^+^ T cells exhibited a substantial delay of cell cycle completion in response to antigen stimulation.

## Materials and Methods

### Animals and Cells

Ezh2 (Ezh2^fl/fl^) mice were generated as described (20) and obtained from MMRRC repository. Cd4Cre was obtained from Taconic, and GzmbCre, OT-I, ROSA-26Sor^tm39(CAG-hop/EYFP)^, and B6.SJL-PtpraJ mice from Jackson Laboratory. Ezh2^fl/fl^ mice were crossed with Cd4Cre to generate the Ezh2^fl/fl^-Cd4Cre^+^ (Ezh2-c-KO) strain. Ezh2^fl/fl^-Cd4Cre mice were further crossed with OT-I to generate Ezh2^fl/fl^-Cd4Cre OT-I (Ezh2-c-KO OT-I) strain. Ezh2^fl/fl^ was also crossed with GzmbCre and Gt(ROSA)26Sor^tm39(CAG-hop/EYFP)Hze^ to create Ezh2^fl/fl^GzmbCre-YFP (Ezh2-g-KO) mice. All mice were maintained under specific pathogen free conditions at the animal facility of National Institute on Aging, and animal care was conducted in accordance with the guidelines of NIH.

CD8^+^ T cells were isolated from splenocytes or blood obtained from different murine strains. Naïve CD8^+^ T cells were defined as CD44^−^CD62L^+^ and purified using StemCell CD8^+^ Naïve T cell isolation kit with a final purity of more than 95%. Memory precursor and central memory CD8^+^ T cells were defined as CD127^+^KLRG1^−^ and CD44^+^CD62L^+^, respectively. Cells were cultured in RPMI-1640 with 10% FBS, 10 mM HEPES, 0.11 nM beta-metcaptoethanol, and 1× Pen/Strep/Glu from Thermo-Fisher. CD8^+^ T cell stimulations performed using plate coated anti-CD3 (2C11, 5 µg/ml) and soluble αCD28 (37.51, 1 µg/ml) (Biolegend).

### *Listeria* Infection

*Listeria monocytogenes* (10403S) with engineered OVA was a gift from Dr. Hao Shen of University of Pennsylvania and cultured in the Brain Heart Infusion (BHI) media with 10 µg/ml erythromycin. Mice were immunized *via* tail vein injections of 5 × 10^4^ cfu of *Listeria monocytogenes* as infection model. All infection experiments were conducted under BSL-2 condition with approved protocol.

### Adoptive Transfer

Naïve CD8^+^ T cells were isolated and purified from the spleens of CD45.2^+^ Ezh2-c-KO or Ezh2-c-KO-OT1 mice and adoptive transferred into CD45.1^+^ B6.SJL-PtpraJ animals. The mice were immunized with *Listeria monocytogenes* on the next day and sacrificed at the indicated time for different experiments or bled at the indicated time for flow cytometric analysis.

### Flow Cytometry Analysis and Cell Sorting

The antibodies used for detection of surface and intracellular molecules such as CD4, CD8, CD45.1, CD45.2, CD127, KLRG1, CD69, CD25, CD44, CD62L, IFNγ, TNFα, IL2, Gzmb, BrdU, and annexin V conjugated to various fluorescent dyes were purchased from Biolegend and OVA-dextramer from Immudex. For activation-induced expression of activation markers, CD8^+^ naïve T cells were stimulated by anti-CD3/CD28 antibodies for 72 h and harvested for surface expression of CD69, CD25, and CD44. For activation-induced cytokine expression, naïve CD8^+^ T cells were stimulated by anti-CD3/CD28 antibodies for 68 h, and PMA (20 ng/ml), Ionomycin (1 µg/ml), and Golgi plug (1 µl/ml) were added and incubated 4 additional hours before proceeded for cytokines and grazyme B analysis. Cells were permeabilized with Fix/Perm buffer (BD bioscience) and blocked with 7% normal rat serum (Stemcell technology) to reduce nonspecific antibody staining. Permeabilized cells were stained in Perm buffer (contains saponin) with cytokine specific or representative isotype control antibodies. Flow cytometry data were acquired on a BD FACSCantoII or Accuri C6 flow cytometer, and the results were analyzed with FlowJo (10.3) (Tree Star).

### Microarray Data Collection and Analysis

Total RNA was extracted from Ezh2-c-KO and WT naïve CD8^+^ T cells after stimulation (0, 16, or 72 h) with RNaeasy Kit on QIAcube (Qiagen) in three biological replications. RNA was labeled using Quick Amp Labeling two-color kit (Agilent) with Cy3 dye used for sample RNA and Cy5 dye for home-made T cell RNA control for all array and hybridized to Agilent-028005 SurePrint G3 Mouse Gene Expression v2 8 × 60K Microarray or Agilent-026655 Whole Mouse Genome v2 4 × 44K Microarray according to the manufacturer’s protocols. The hybridized chips were scanned with SureScan Microarray Scanner D (Agilent), and the expression level was determined with Agilent Feature Extraction software 11.5.1.1. Microarray data are submitted to GEO/NCBI database, accession number GES106426. Data were log-transformed and then normalized based on Cy5 signal intensity. Data from 60K and 44K microarray platforms were combined using batch normalization by equalizing median log-transformed gene expression values for seven samples that were hybridized to both array platforms. To avoid redundancy, these seven samples were further analyzed using only 60K data. The microarray data were further analyzed with ExAtlas (NIA, NIH) and BRB Array Tools (NCI, NIH).

### Quantitative Reverse Transcription Polymerase Chain Reaction

Total RNA was extracted with RNaeasy Kit on QIAcube (Qiagen) from naïve CD8^+^ T cells. RNA was reverse transcribed to cDNA using Super Script III (Invitrogen). Quantitative PCR was performed with SYBR green (Qiagen) by standard protocol. The probes and primers used for PCR are listed in Table S3 in Supplementary Material.

### Proliferation Assay

Proliferation assays were carried out with CFSE and BrdU incorporation assay. For CFSE incorporation assay, naïve CD8^+^ T cells were labeled with 1 µM CFSE or CellTrace Violet (Invitrogen) according to the manufacturer’s instructions and then stimulated with anti-CD3/CD28. The labeled cells were collected, and flow cytometric analysis was performed at different time points, with or without counting beads (Spherotech). For BrdU assay, naïve CD8^+^ T cells were first stimulated with anti-CD3/CD28. One hour before the measurement, the activated cells were labeled with 10 µM BrdU (Sigma) and stained with surface markers, intracellular αBrdU antibodies and 7-AAD. Data were acquired on BD FACSCantoII and analyzed with FlowJo (Tree Star).

### Apoptosis Assay

Apoptosis assays were performed with annexin V staining, caspase 3/7 staining, and TUNEL assay. For annexin V staining, prepared cells were first stained with cell surface antibodies and then with fluorophore-conjugated annexin V and 7-AAD in the annexin V staining buffer. The cells were acquired immediately on a flow cytometer. Caspase 3/7 staining was performed with Vybrant FAM Caspase-3 and -7 Assay Kit (Thermo-Fisher) according to the manufacturer’s protocols. Briefly, cells were incubated with FLICA working solution 1 h before measurement and stained with other fluorescent conjugated cell surface antibodies and 7-AAD. The stained cells were acquired immediately on a flow cytometer. The TUNEL assay was performed with APO-BrdU TUNEL Assay Kit (Thermo-Fisher) according to the manufacturer’s protocol. Briefly, cells were fixed with 1% (w/v) paraformaldehyde in PBS on ice for 15 min, and then resuspended in ice-cold 70% ethanol and further incubated on ice for a minimum of 30 min. The fixed cells were labeled with BrdU with TdT enzyme for 60 min, and then stained with fluorescent-conjugated anti-BrdU antibody. The cells were acquired on a flow cytometer and analyzed as mentioned earlier.

### Chromatin Immunoprecipitation

Naïve CD8^+^ T cells were isolated freshly and stimulated with anti-CD3/CD28 *in vitro* for 72 h. The cells were digested by 0.2U MNase per million cells for 10 min and sonicated (Diagenode) with a power setting of 5 for 4 repeats of 30 s followed by 30 s break between each repeat in ice-cold water to obtain the average size between 100 and 300 bps. The sonicated products were dialyzed with 10000 MWCO Cassette G2 (Thermo-Fisher) in 400 ml RIPA buffer for 2 h at 4°C and then incubated overnight with 2 µg anti-H3K27me3 antibody (Millipore, 07-449) or IgG-conjugated Dynabeads Protein G (Thermo-Fisher). The beads were washed with RIPA, LiCl, and TE buffer sequentially and recovered overnight at 65°C with TE supplemented with 10% SDS and proteinase K (MBiotech). The ChIP DNA was purified with phenol/chloroform (Invitrogen) and precipitated for quantitative PCR. The primers used for ChIP are listed in Table S3 in Supplementary Material. The specific H3K27me3 ChIP was calculated by subtracting the value of IgG (% of H3K27me3 in input − % of IgG in input). We also used the primer covering non-promoter region of these gene loci (P0) as additional control. The P0 regions were selected based on published H3K27me3 ChIP-seq data from other cell lines.

### Microscopy and Image Analysis

Naïve CD8^+^ T cells were isolated and labeled with CellTrace Far Red in Phenol Red-free RPMI-1640 media. 35-mm dishes were coated with anti-CD3 and a 4-well silicone micro-insert (ibidi) placed in the same dish to hold the cells. Cells to be imaged were mixed with soluble anti-CD28 and pipetted into the micro-inserts. Cells were also plated at a density of 1 × 10^6^ in the surrounding area of the insert. The insert was fully submerged by adding additional Phenol Red-free RPMI-1640 media. Cells were imaged on a Zeiss LSM880 platform. Image acquisition was performed using the Tile scan (6 × 4 or 5 × 5) feature of the Zen 2.3 (black) suite. The following parameters were set up for long-term image acquisition of CellTrace Far Red dye along with DIC using a Plan-Apochromat 40×/1.4 Oil DIC M27 objective—pixel width: 0.692 µm (512 × 512 pixels), 8-bit depth, line sequential scan, zoom: 0.6, averaging: 1, excitation wavelength: 633 nm, detection wavelength: 633–695 nm, laser power: 0.0200%, binning mode: 1 × 1, detector gain (850 V for far red, 750 V for DIC), digital gain: 0.30, offset: 0, pinhole: 600 µm (max). Definite Focus setup (every tile scan, every time point) was used to maintain the focus during image acquisition. Images were acquired at 10 min interval for 24–48 h in an incubation chamber maintained at 37°C with 5% CO_2_. Composite images were further stitched in the same suite and exported as TIFF for analysis. Acquired images were analyzed on Fiji. Cells and their divisions were tracked manually.

### Ezh2 Genotyping at Single Cell Level

Naïve CD8^+^ T cells from WT, Ezh2-c-KO, or Ezh2-g-KO mice were isolated, labeled with CFSE, and stimulated with anti-CD3/CD28 for 72 h *in vitro*. The undivided and high divided (>3 division) stimulated cells were sorted into a 96-well plate containing 1× PCR reagents. The sorted plates were immediately spin down at 1,400 rpm for 5 min and heated at 95°C for 5 min, following with Ezh2 genotyping PCR and checked with gel electrophoresis. The primers used for Ezh2 genotyping were listed in Table S3 in Supplementary Material.

### Statistical Analysis

Unless specifically indicated, *p* values were calculated using Student’s *t*-test. ANOVA test was used for comparison of multiple samples. *, **, and *** represent *p* < 0.05, 0.01, and 0.001, respectively.

### Accession Numbers

Microarray data were available in the Gene Expression Omnibus (GEO) database (http://www.ncbi.nlm.nih.gov/gds) under GES106426 (GSE106424 and GSE106425).

## Results

### Ezh2-Deficient Naïve CD8^+^ T Cells Have Diminished Activation-Induced Expansion and Differentiation During *Listeria* Infection

**Figure 1:**
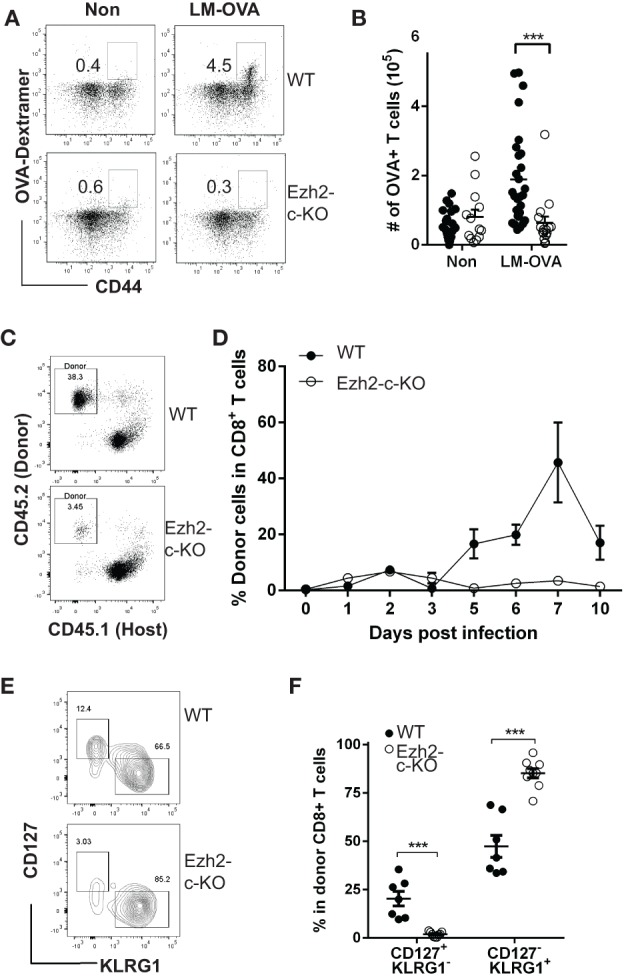
Defects in expansion and differentiation of naive CD8^+^ T cell in response to *Listeria* (LM-OVA) infection in the absence of Ezh2. **(A)** Representative plot of OVA-specific CD8^+^ T cells from spleen before (Non) and after LM-OVA infection. Wild-type (Ezh2^fl/fl^-Cd4Cre^−^ as WT) or Ezh2^fl/fl^-Cd4Cre^+^ (Ezh2-c-KO) mice were injected 5 × 10^4^ cfu LM-OVA by i.v. Number of OVA-specific CD8^+^ T cells from spleen were analyzed on 7 days post infection. OVA_257–264_ dextramer was used for identification of OVA-specific CD8^+^ T cells. **(B)** Number of OVA-specific CD8^+^ T cells in WT and Ezh2-c-KO mice before and 7 days after LM-OVA infection (experiments = 4, 2–7 mice/experiment). **(C)** Representative dot plot of donor blood CD8^+^ T cells in host at day 7 after LM-OVA infection. Naïve CD8^+^ T cells (10^5^) were isolated from WT OT-I or Ezh2-c-KO OT-I mice and adoptively transferred into congenic mice (CD45.1) and 5 × 10^4^ cfu LM-OVA were injected i.v next day. Donor cells in blood were measured using flow cytometry at day 1–10 post infection. **(D)** Average percentages of donor CD8^+^ T cells during the 10-day course of LM-OVA infection (data were averaged from 5 to 6 mice of each time point) from WT and Ezh2-c-KO mice. Mean and SEM are presented here and used in all figures. **(E)** Representative plot of memory precursors (CD127^+^KLRG1^−^) and short live effector cells (CD127^−^KLRG1^+^) at day 12 post infection from WT and Ezh2-c-KO mice. **(F)** Percentage of memory precursors and short live effector cells at day 12 post infection. *p < 0.05, **p < 0.01, ***p < 0.001 based on Student’s *t*-test and used in all figures.

To determine the roles of Ezh2 in naïve CD8^+^ T cell function, we used Ezh2 conditional knockout mice (Ezh2^fl/fl^-Cd4Cre^+^ referred as Ezh2-c-KO, and both Ezh2^fl/fl^-Cd4Cre^−^ and Ezh2WT-Cd4Cre^+^ as WT) (20) and challenged with *Listeria*-OVA (LM-OVA) (31). Although both total CD8^+^- and OVA-specific CD8^+^ T cells from WT mice increased significantly in response to *Listeria* infection, little to no expansion of OVA(OVA_257-264_)-specific CD8^+^ T cells was observed in Ezh2-c-KO mice (Figures 1A,B). The deletion of Ezh2 in CD8^+^ T cells from Ezh2-c-KO mice was completed (Figure S1 in Supplementary Material). To rule out potential differences in the number of OVA-specific CD8^+^ T cells between WT and Ezh2-c-KO mice, we adoptively transferred the same number of isolated CD8^+^ T cells (1 × 10^5^) from OT-I or Ezh2-c-KO OT-I mice into the congenic CD45.1 mice followed by LM-OVA infection. Again, no detectable expansion of OVA-specific CD8^+^ T cells was observed in Ezh2-c-KO mice throughout 10-day post infection period, while OVA specific CD8^+^ T cells from OT-I mice had significant expansion at day 5, peaked at day 7, and reduced at day 10 (Figures 1C,D). In addition to lack of expansion, CD8^+^ T cells from Ezh2-c-KO mice were all effector cells (CD127^−^KLRG1^+^) with little memory precursor cells (CD127^+^KLRG1^−^) at day 12 post LM-OVA infection (Figures 1E,F). These results demonstrate a role of Ezh2 in activation-induced expansion and differentiation of CD8^+^ T cells.

### Ezh2-Deficient CD8^+^ T Cells Are Activated but Undergo Increased Apoptosis

**Figure 2:**
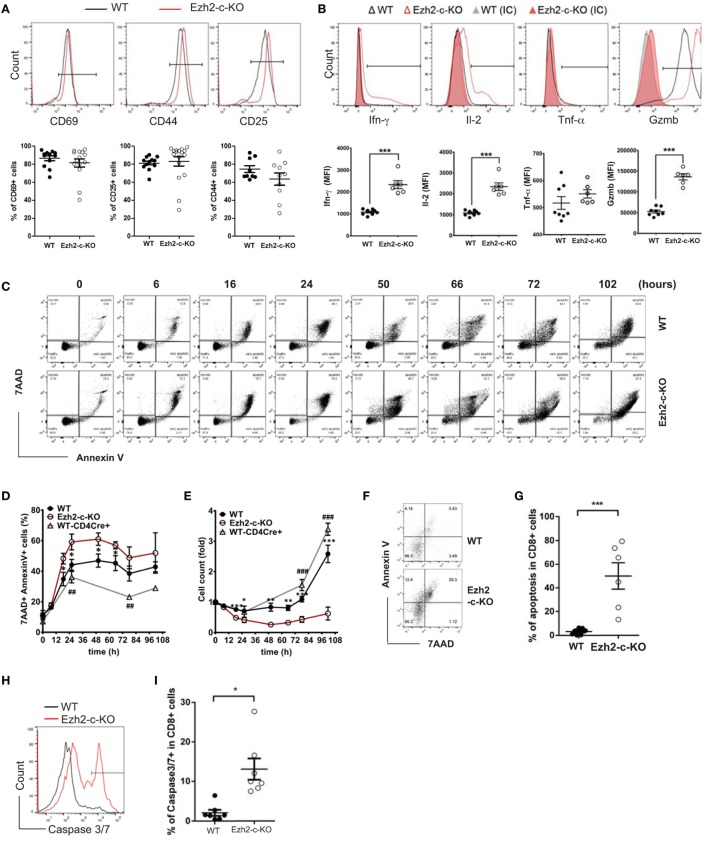
Ezh2-deficient naïve CD8^+^ T cells express activation markers but undergo increased apoptosis in response to stimulation. **(A)** Representative histogram and summary of activation marker CD69, CD25, and CD44 in activated naïve CD8^+^ T cells from WT and Ezh2-c-KO mice. Naive CD8^+^ T cells were isolated from spleen of WT or Ezh2-c-KO mice and stimulated with anti-CD3 (5μg/ml)/CD28 (1 µg/ml) antibodies for 72 h. Each dot represents each mouse and experiments repeated four times and each with 2–3 mice. **(B)** Representative histogram and summary of Ifn-γ, Il-2, Tnf-α, and Granzyme-B (Gzmb) in activated naïve CD8^+^ T cells from WT and Ezh2-c-KO mice. After 68 h of stimulation with anti-CD3/CD28 antibodies, PMA (20 ng/ml), Ionomycin (1 µg/ml), and Golgi plug (1 µl/ml) were added for another 4 h. Each dot represents each mouse and experiments repeated four times and each with 2–3 mice. **(C–E)** Increased apoptosis in *in vitro* activated naive CD8^+^ T cells in the absence of Ezh2. Naïve CD8^+^ T cells were isolated from spleen of WT (both Ezh2^fl/fl^Cd4Cre− and Ezh2^+/+^Cd4cre+) or Ezh2-c-KO mice and stimulated with anti-CD3/CD28 *in vitro*. Apoptosis was analyzed by annexin V and 7-AAD at the indicated time points by flow cytometry. Experiments were repeated 2–3 times, 9–14 mice per each time **(C)**. Representative dot plots of annexin V staining. **(D)** Average percentages of apoptotic cells from E. **(E)** Average numbers of live cells. **(F–I)** Increased apoptosis in activated CD8^+^ T cells *in vivo* in Ezh2-c-KO mice. WT or Ezh2-c-KO mice were infected with 5 × 10^4^ cfu LM-OVA for 7 days. Splenocytes were isolated and stained with Annexin V/7AAD and CD8. **(F)** Representative plot of Annexin V and 7-AAD staining in CD8^+^ T cells. **(G)** Summary of data from **(F)** (experiments were repeated twice, 3 mice each time). **(H)** Histogram of Caspase 3/7 staining. **(I)**. Summarized of data from **(H)** (experiments were repeated twice, 3–4 mice each time).

To further investigate the defects in the absence of Ezh2, CD8^+^ naïve T cells from Ezh2-c-KO and WT mice were isolated and activated *in vitro* with anti-CD3 and anti-CD28 antibodies (anti-CD3/CD28). We examined the expression of three activation markers (CD69, CD25, and CD44), three cytokines (Ifn-γ, Il-2, and Tnf-α), and one effector molecule (Granzyme B) after 72 h of stimulation. We found no significant differences in expression of three activation markers between Ezh2-c-KO and WT mice (Figure 2A). In agreement with increase effector cells (CD127^−^KLRG1^+^), CD8^+^ naïve T cells from Ezh2-c-KO mice had significantly higher MFI of Ifn-γ, Il-2, and Granzyme B than that from WT mice (Figure 2B). These findings show that Ezh2-deficient CD8^+^ T cells have no defects in *in vitro* activation-induced expression of activation markers but enhanced expression of two cytokines (Ifn-γ and Il-2) and one effector molecule (Gzmb).

We further observed significantly reduced number of live cells after stimulation as a consequence of increased death of Ezh2-deficient CD8^+^ T cells. First, there was a significant increase in apoptotic cells defined by annexin V^+^ and 7AAD^+^ 16 h after stimulation (Figures 2C–E). Increased apoptosis in activated Ezh2-deficient CD8^+^ T cells was also observed with the increase in DNA fragmentation by TUNEL assay (Figure S2 in Supplementary Material). Consequently, there were significantly fewer numbers of CD8^+^ T cells from Ezh2-deficient mice than from WT mice (WT and Ezh2WT-Cd4cre^+^) after *in vitro* stimulation (Figure 2E). To determine whether increased apoptosis occurred under *in vivo* conditions, we used adoptive transfer with LM-OVA infection model and found that Ezh2-deficient CD8^+^ T cells had more apoptosis (Figures 2F,G) and higher percentage of cells expressing key apoptotic activators, caspase 3/7, than WT CD8^+^ T cells (Figures 2H,I). Together, these findings demonstrate that Ezh2-deficient CD8^+^ T cells have normal response to activation-induced expression of key functional molecules but significantly increased apoptosis.

### Ezh2-Deficient Naïve CD8^+^ T Cells Have Impaired Activation-Induced Proliferation

**Figure 3:**
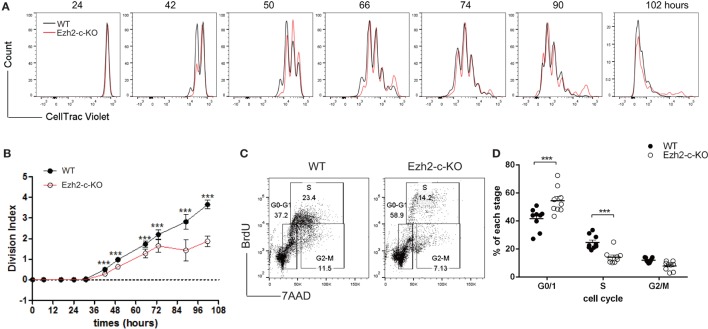
Defects in proliferation of Ezh2 deficient CD8^+^ T cells. **(A–D)** CellTrace Violet labeled WT or Ezh2-c-KO CD8^+^ naïve T cells were stimulated with anti-CD3 and anti-CD28 for up to 102 h *in vitro*. Representative cell division was monitored **(A)** and Division Index was calculated **(B)**. Four repeats, 3–6 mice each time at indicated time points. **(C,D)** WT or Ezh2-c-KO CD8^+^ naïve T cells were stimulated with anti-CD3/CD28 for 48 h and BrdU was added into culture for 1 h before harvest. Cell cycle was analyzed with anti-BrdU antibody and 7-AAD staining [representative in **(C)**] and percentages of each stage were summarized in **(D)** gated on 7-AAD^−^ cells (3 repeats, 2–3 mice each time).

To further determine if the reduction in the number of Ezh2-deficient naïve CD8^+^ T cells post-activation was also influenced by a reduction in cell proliferation, we used the CellTrace fluorescent dye incorporation assay and found that Ezh2-deficient CD8^+^ T cells had more undivided cells as shown by a decrease of Division Index (compared divided cells over undivided cells) after stimulation *in vitro* compared to WT naïve CD8^+^ T cells (Figures 3A,B). We then analyzed the cell cycle progression after activation using the BrdU incorporation assay and found that Ezh2-deficient CD8^+^ T cells were fewer in S-phase but more in G0/G1 phase compared to WT cells (Figures 3C,D). These results indicate that Ezh2-deficient CD8^+^ T cells have impaired cell cycle progression from G0/G1 to S phase in response to stimulation.

### Ezh2-Deficient CD8^+^ T Cells Have Altered Expression of Apoptosis and Cell Cycle Related Genes

**Figure 4:**
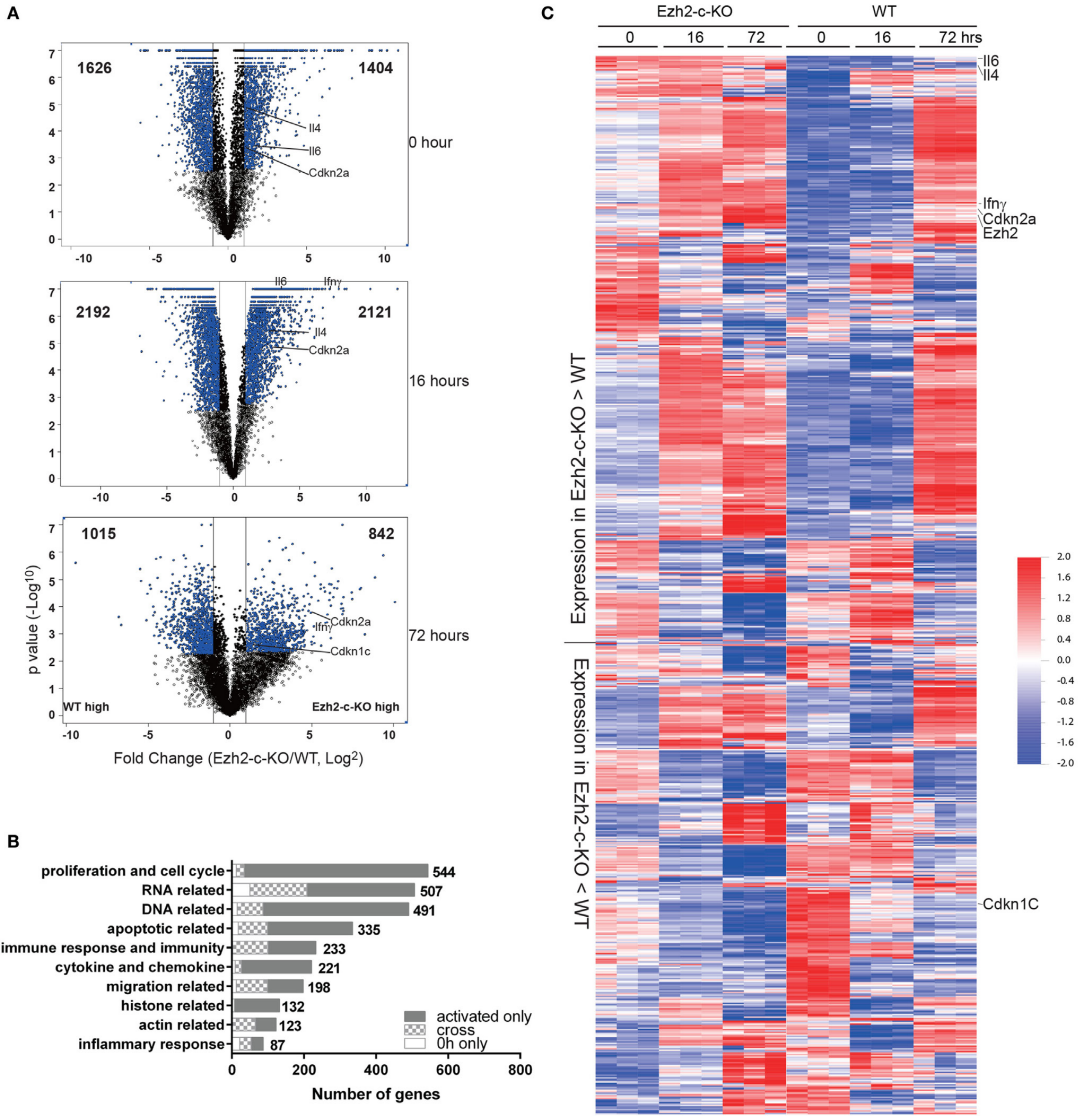
Altered expression of genes related to cell cycle regulation and apoptosis in Ezh2-deficient CD8^+^ T cells post-activation. **(A–C)** Freshly isolated naïve CD8^+^ T cells from WT or Ezh2-c-KO mice as well as stimulated with anti-CD3/CD28 *in vitro* for 16 and 72 h were analyzed by gene expression using Agilent microarray method. Each group had three independent biological repeats. **(A)** Volcano plot of altered expression of genes having more than twofold changes (FDR < 0.05) between WT and Ezh2-c-KO at 0, 16, and 72 h. The up or down expressed genes were presented on right and left with the indicated number, respectively. A few selected significantly changed genes were marked. **(B)** The altered genes were analyzed with GO between WT and Ezh2-c-KO groups at 0, 16, and 72 h, respectively. The top 10 GO categories were summarized with the altered genes at 0 h only, activated (16 and 72 h) only and the cross between them. **(C)** Heatmap of all altered genes increased in Ezh2-c-KO mice and increased in WT mice. The interested genes were highlighted on the side of the heatmap. The scale of the data is in log_10_.

To further investigate the mechanism of the observed defects in Ezh2-deficient CD8^+^ T cells, we analyzed the transcriptome by microarray of naïve CD8^+^ T cells from Ezh2-c-KO and WT mice at three different time points: freshly isolated and *in vitro* stimulated by anti-CD3/CD28 for 16 and 72 h, respectively. We observed 1,626 and 1,404 genes with increased and decreased expression (>2-fold change and FDR < 0.05) in freshly isolated Ezh2-c-KO naïve CD8^+^ T cells compared to WT cells, respectively. After stimulation, there were 2,192 and 1,015 genes with increased expression and 2,121 and 842 genes with decreased expression (Ezh2-c-KO vs. WT) at 16 and 72 h, respectively (Figure 4A). Further functional analysis suggested that freshly isolated naïve CD8^+^ T cells from Ezh2-c-KO mice had altered expression in RNA related, DNA related, apoptosis, immune response, inflammatory response, actin and migration related biological processes. After stimulation, additional alteration in proliferation, cytokines and chemokines, histone-related biological processes was observed (Figure 4B). This indicates that Ezh2 deficiency affects a wide range of molecular and cellular function of naïve CD8^+^ T cells in a temporal manner (Tables S1 and S2 in Supplementary Material).

We then focused on apoptosis and cell cycle related genes which may explain the cellular defects we observed in the Ezh2-c-KO mice. Among 335 increased apoptosis related genes found in Ezh2-c-KO mice, 99 were detected in freshly isolated naïve CD8^+^ T cells (Figures 4B,C). As Ezh2 deletion occurs during T cell development in thymus in the Ezh2-c-KO mice, mature naïve CD8^+^ T cells in periphery had already carried these defects and activation further exuberated the apoptosis process. In contrast, the expression of most cell cycle checkpoint genes were not significantly different between Ezh2-deficient and WT naïve CD8^+^ T cells but several cycle checkpoint genes including *Cdkn2a* and *Cdkn2b* were significantly increased after 72 h post-activation in Ezh2-deficient CD8^+^ T cells compared to WT CD8^+^ T cells (Figures 4B,C). Together, the transcriptome analysis provides evidence of altered gene expressions that is responsible for activation-induced increased apoptosis and cell cycle defects in the absence of Ezh2.

### Ezh2 Regulates H3K27me3 of *Cdkn1c* and *Cdkn2a* Locus

**Figure 5:**
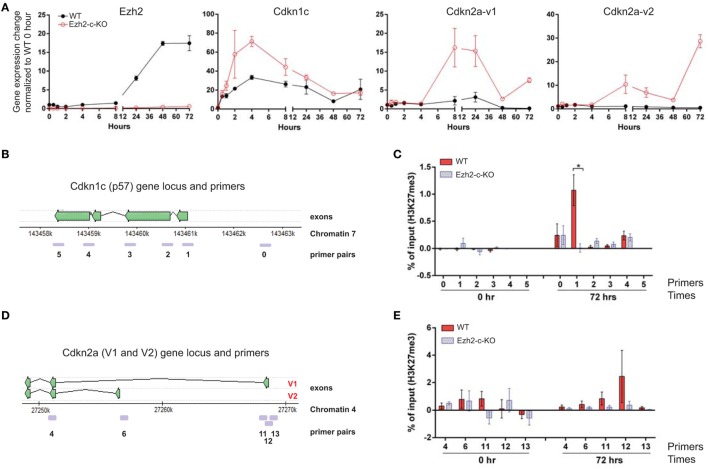
Repression of *Cdkn1c* and *Cdkn2a* expression during naïve CD8^+^ T cells activation by Ezh2. **(A)** Naïve CD8^+^ T cells were isolated from spleen of WT or Ezh2-c-KO mice and stimulated with anti-CD3/CD28 *in vitro*. Cells were collected at 0, 0.5, 1, 2, 4, 8, 24, 48, and 72 h after stimulation for gene expression analysis. Relative levels of *Cdkn1c* (*p57*) and *Cdkn2a* (*v1 and v2*) were determined by real time RT-PCR and normalized to RPL32 and represented as to WT-unstimulated naïve CD8^+^ T cells. **(B–E)** Measurement of H3K27me3 levels at *Cdkn1c* and *Cdkn2a* gene loci by Chromatin immunoprecipitation-PCR. **(B)** *Cdkn1c* gene locus and primer locations. **(C)** Relative amount of H3K27me3 in specific locations of *Cdkn1c* locus in freshly isolated (0) and stimulated (72 h) naïve CD8^+^ T cells of WT or Ezh2-c-KO mice. **(D)** *Cdkn2a v1* and *v2* gene locus and primer locations. **(E)** Relative amount of H3K27me3 in specific locations of *Cdkn2a* locus in freshly isolated (0) and stimulated (72 h) naïve CD8^+^ T cells of WT or Ezh2-c-KO mice. Data were present as “% of H3K27me3 in input − % of IgG in input.” The experiments were repeats six times.

To understand how early the changes in expression of cell cycle related-genes happens in the absence of Ezh2, we included four additional early time points (1, 2, 4, and 8 h after stimulation) during the course of 72 h after anti-CD3/CD28 stimulation. Ezh2 mRNA levels were increased at 8 h, peaked around 48–72 h after stimulation (Figure 5A). We then compared expressions of 12 cell-cycle regulators and found that *Cdkn1c* (p57) was dramatically increased at 2 h and peaked at 4 h (>2-folds higher in Ezh2-deficient CD8^+^ T cells than in WT CD8^+^ T cells) after stimulation (Figure 5A). For *Cdkn2* family, expression of *Cdkn2aV1* (*Arf*) and *Cdkn2aV2* (*p16*), key regulators for apoptosis and cell cycle, respectively, were increased in Ezh2-deficient naïve CD8^+^ T cells compared to WT naïve CD8^+^ T cells from 8 to 72 h after stimulation (Figure 5A). *Cdkn2b* (p15) was also expressed higher in Ezh2 deficient than in WT naïve CD8^+^ T cells from 8 to 16 h (Figure S3 in Supplementary Material). However, we did not find significant differences in gene expressions of either cell cycle activators such as *Ccnd2, Ccnd3, Cdk2, Cdk4, Cdk6, E2F1, c-Myc*, and *Gadd45a* or cell cycle inhibitors *Cdkn1a* (*p21*) and *Cdkn1b* (*p27*) between Ezh2-deficient and WT CD8^+^ T cells of Figure S3 in Supplementary Material. Together, these findings show that Ezh2 may regulate expression of *Cdkn1c, Cdkn2aV1* (*Arf*), and *Cdkn2aV2* (*p16*) in naïve CD8^+^ T cells.

To further determine whether the enhanced expressions of *Cdkn1c, Cdkn2aV1* (*Arf*) and *Cdkn2aV2* (*p16*) were the consequence of loss of Ezh2, we analyzed H3K27me3 in these genomic loci using ChIP assay. We found that H3K27me3 was present in the promoter region of *Cdkn1c* locus (Figures 5B,C). The H3K27me3 occupancy was higher at the promoter region of exon 1a, but not exon 1b (Figures 5D,E) which indicates that naïve CD8^+^ T cells activation induce more repression of *Cdkn2aV1* rather than repressing *Cdkn2aV2*. These data suggest that Ezh2-mediated H3K27me3 regulates *Cdkn1c, Cdkn2aV1* (*Arf*), and *Cdkn2aV2* (*p16*) expressions during naïve CD8^+^ T cell activation.

### Immediate Deletion of Ezh2 Affects Activation-Induced Cell Cycle but Not Apoptosis in CD8^+^ T Cell

**Figure 6:**
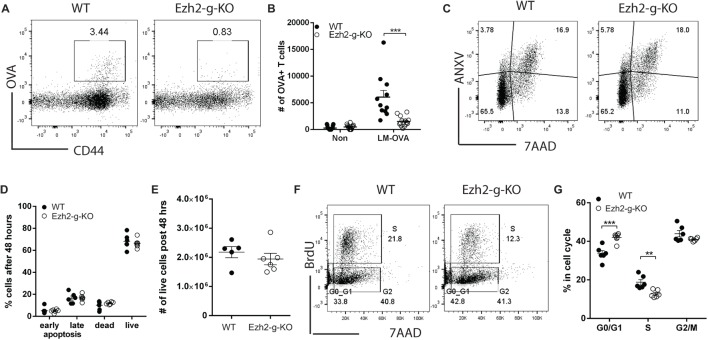
Defects in activation-induced proliferation but not apoptosis of naïve CD8^+^ T cells from Ezh2-g-KO mice. **(A,B)** Failed OVA-specific CD8^+^ T cell differentiation in Ezh2-g-KO mice. WT or Ezh2-g-KO mice were infected with 5 × 10^4^ LM-OVA, and OVA-specific CD8^+^ T cells were detected at 7 days after infection. **(A)** Representative staining of OVA-specific CD8^+^ T cells with OVA_257-264_-dextramer. **(B)** Percentages of OVA-specific CD8^+^ T cells from multiple mice (experiments were repeated four times with 2–7 mice each time). **(C–E)** Apoptosis analysis. Naïve CD8^+^ T cells were isolated from spleen of WT or Ezh2-g-KO mice, labeled with CellTrace Violet dye and stimulated with anti-CD3/CD28 *in vitro* for 4 days. Cells were harvested and stained with ANXV and 7AAD. Representative plots are presented in **(C)** and percentages are summarized in **(D)**. Numbers of live cells were summarized in **(E)**. The experiments were repeated three times, with 2–3 mice each repeat. **(F,G)** Naïve CD8^+^ T cells from WT or Ezh2-g-KO were stimulated with anti-CD3/CD28 for 48 h, and BrdU was added into culture for 1 h before harvest. Cell cycle was analyzed [representative in **(F)**] and percentages were summarized in **(G)** (3 repeats with 3 mice each time).

The deletion of Ezh2 in Ezh2^fl/fl^Cd4Cre^+^ mice happened at double positive thymocyte stage and naïve CD8^+^ T cells of spleen had enhanced expression of apoptosis-related genes. To investigate the immediate effects of losing Ezh2, we crossed Ezh2^fl/fl^ with GzmbCre and Gt(ROSA)26Sor^tm39(CAG-hop/EYFP)Hze^. The deletion of Ezh2 took place only after activation of T cells which turned on GzmbCre hence leading to Ezh2 deletion and EYFP expression (Ezh2^fl/fl^GzmbCre^+^ as Ezh2-g-KO and Ezh2^fl/fl^ GzmbCre^−^ as WT) (Figure S4 in Supplementary Material). We then used Ezh2-g-KO mice to determine the immediate effects of deleting Ezh2 in naïve CD8^+^ T cell differentiation *in vivo*. Ezh2-g-KO and WT (Ezh2^fl/fl^ GzmbCre^−^) mice were infected with LM-OVA, and OVA-specific CD8^+^ T cells were measured at day 7 after immunization. Comparable to the Ezh2-c-KO mice, Ezh2-g-KO mice also showed a marked defect in CD8^+^ T cell differentiation after LM-OVA infection *in vivo* (Figures 6A,B). However, unlike Ezh2-c-KO mice, CD8^+^ T cells of Ezh2-g-KO mice had comparable percentages of live, apoptosis, and dead cells with CD8^+^ T cells of WT mice after *in vitro* stimulation (Figures 6C,D) and total live cells (Figure 6E). In contrast, we observed significantly decreased percentage of cells in S-phase and more in G0/G1 phase in naïve CD8^+^ T cells of Ezh2-g-KO mice compared to that of WT (Figures 6F,G). Together, these findings indicate that regulation of cell cycle and apoptosis by Ezh2 are temporally separated events and loss of Ezh2 first affects cell cycle progression in naïve CD8^+^ T cells.

**Figure 7:**
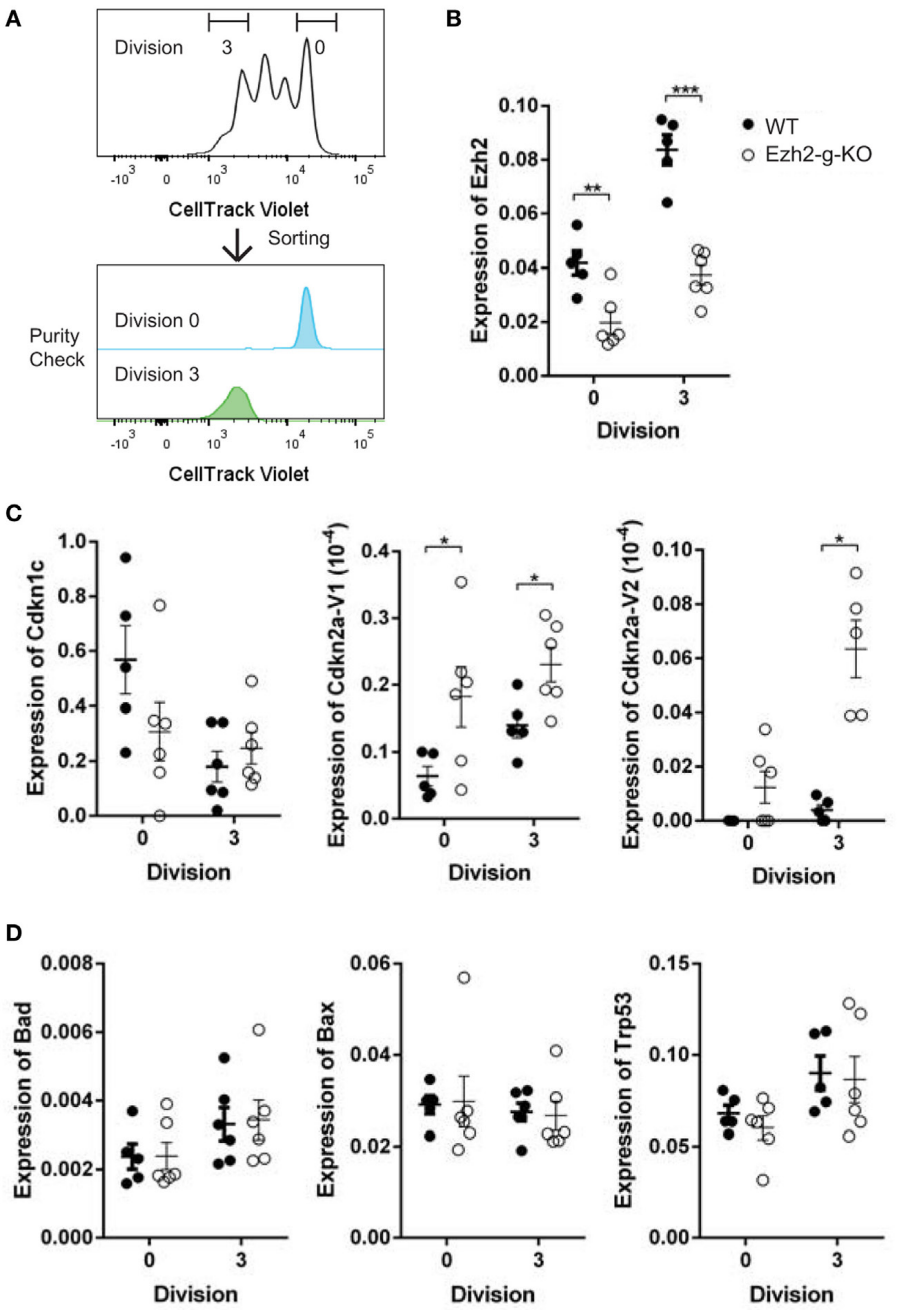
Increased expression of Cdkns but not apoptosis-related in stimulated naïve CD8^+^ T cells from Ezh2-g-KO mice. Naïve CD8^+^ T cells were isolated from WT or Ezh2-g-KO mice and labeled with CellTrace Violet and stimulated with anti-CD3/CD28 for 72 h. The undivided (Division 0) and high divided (≥Division 3) cells were sorted, and the expression of selected *Cdkns* and apoptosis genes were measured by real time RT-PCR. **(A)** Cell sort scheme. **(B–D)** Relative amount of mRNA is presented after normalization to *RPL32* including *Ezh2, Cdkn1c, Cdkn2a*, and apoptosis related genes (*Bad, Bax, Trp53*). The experiments were repeated twice, with three mice of each repeat.

To determine whether altered transcriptional changes of cell cycle regulators and apoptosis related genes in naïve CD8^+^ T cells observed from Ezh2-c-KO mice were also found in the Ezh2-g-KO mice, we sorted activated naïve CD8^+^ T cells from WT or Ezh2-g-KO mice into undivided and highly divided (more than three divisions) populations after 72 h of stimulation (anti-CD3/CD28) *in vitro* to allow GzmbCre expression (Figure 7A). We found that both undivided and high divided naïve CD8^+^ T cells showed significantly decreased Ezh2 levels from Ezh2-g-KO compared to the WT mice (Figure 7B). Furthermore, expression of *Cdkn2aV1* and *Cdkn2aV2* were significantly increased, but no change in expression of *Cdkn1c* and apoptotic related genes could be observed in naïve CD8^+^ T cells from Ezh2-g-KO compared to that from WT mice (Figures 7C,D).

### Naïve CD8^+^ T Cells Take Longer to Complete Cell Division in Response to Activation After Ezh2 Deletion

**Figure 8:**
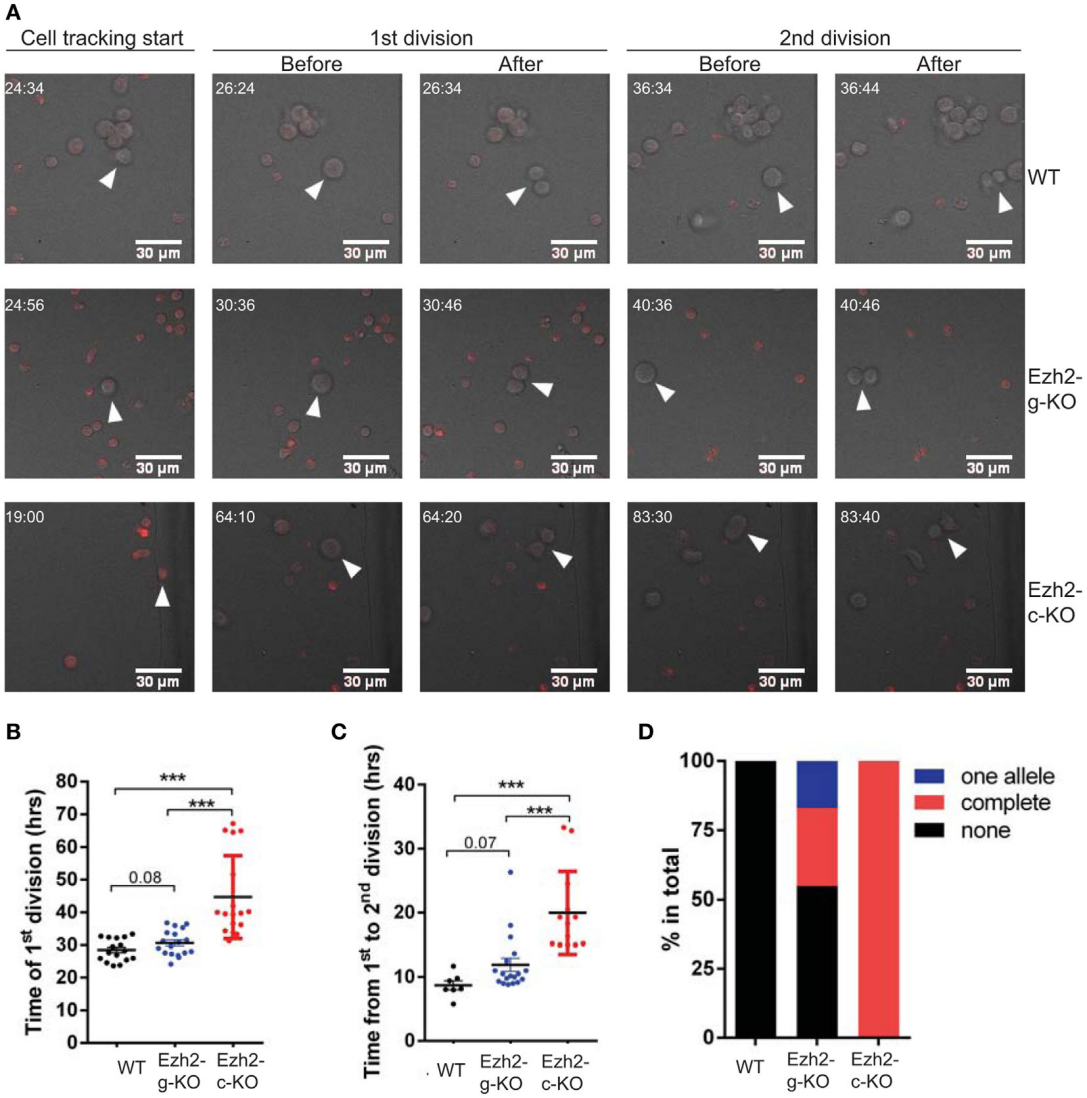
Direct measurement of cell division with real-time live imaging. Naïve CD8^+^ T cells were isolated from Ezh2-g-KO and Ezh2-c-KO mice and labeled with CellTrace Far Red, stimulated with anti-CD3/CD28 and imaged as described in Section “Materials and Methods.” **(A)** Representative images indicating the time points (hh:mm) of the start of cell tracking, the first and second cell divisions, respectively. Also see Videos S1–S3 in Supplementary Material. **(B)** Observed time of the first division (hours) of stimulated naïve CD8^+^ T cells obtained from WT, Ezh2-g-KO, and Ezh2-c-KO mice. **(C)** Observed time of the second cell division of stimulated naïve CD8^+^ T cells. The number on the graphs indicates the *p*-value obtained from Student’s *t*-test. **(D)** Deletion of Ezh2 in activated naïve CD8^+^ T cells from Ezh2-g-KO mice by single cell genotyping. Activated single naïve CD8 T cell from WT, Ezh2-c-KO, and Ezh2-g-KO mice were sorted into 96-well plate (one cell/well). The deletion of Ezh2 was examined as described in Section “Materials and Methods.” WT had 36 single cells (no deletion), Ezh2-c-KO had 38 single cells (complete deletion), and Ezh2-g-KO had 54 single cells with complete deletion (*n* = 15), partial deletion (*n* = 9), and no deletion (*n* = 29) of Ezh2 allele are presented as percentage.

The increased G0/G1 and reduced S phase of naïve CD8^+^ T cells from Ezh2-g-KO mice suggested that immediate deletion of Ezh2 altered activation-induced cell cycle. To directly test this, we developed a method to track naïve CD8^+^ T cell division after anti-CD3/CD28 stimulation in real-time using live cell imaging (Videos S1–S3 in Supplementary Material). We used 2.0 mm × 1.5 mm silicone inserts to culture activated naïve CD8^+^ T cells and imaged the entire area of cultured cells to efficiently track the motile cells (Figure S5 in Supplementary Material). We found that the first division of WT CD8^+^ T cells occurred at an average of 28.4 h (ranging from 23.6 to 33.4 h) after stimulation, which was little faster than that reported for CD4^+^ T cells using CFSE assay (32, 33). Naïve CD8^+^ T cells from Ezh2-c-KO mice had a significantly slower first cell division (44.7 ± 3.1 h) than that of WT mice (28.4 ± 0.9 h). The first cell division time of naïve CD8^+^ T cells from Ezh2-g-KO mice was also slower (mean 30.6 h, range from 24.1 to 36.8 h) than WT mice (Figures 8A,B). Using live imaging, we could also observe the second round of cell division. Interestingly, the average time for second division of WT CD8^+^ T cells was 8.7 h (ranging from 5.8 to 11.7 h) (Figure 8C), which was also faster than the reported 10 h (ranging from 6 to 12 h) in CD4^+^ T cells (32, 33). Again, the average time of second division of CD8^+^ T cells from Ezh2-g-KO mice was 11.9 h (range from 8.8 to 26.3 h) and from Ezh2-c-KO mice was 20 h (Figure 8C). Because we detected lower Ezh2 mRNA (Figure 7B) and the division time of some CD8^+^ T cells from Ezh2-g-KO mice had similar time as that of CD8^+^ T cells from WT mice, we suspected that the complete deletion of Ezh2 in Ezh2-g-KO mice may not have occurred. We further performed single cell genotyping of Ezh2 in naïve CD8^+^ T cells of these mice after *in vitro* stimulation and found that Ezh2 was completely deleted in 28.3% cells, deletion of one allele happened in 17% of cells, and the remaining 54.7% of cells had no deletion (Figure 8D). Thus, the delay of cell cycle completion would have been more severe had the deletion taken place in those 54.7% cells of the Ezh2-g-KO mice. Taken together, we tracked highly motile CD8^+^ T cells through their division events in real time after activation and demonstrated the precise time delay of cell cycle in Ezh2 deleted naïve CD8^+^ T cells.

## Discussion

Ezh2 catalyzes H3K27 trimethylation to repress genes involving multiple critical biological processes in development and differentiation of T cells. In this study, we demonstrated that the role of Ezh2 in regulation of cell proliferation and apoptosis are two temporally separable events during naïve CD8^+^ T cell activation. Naïve CD8^+^ T cells from spleen of Ezh2-c-KO mice had altered expression of cell cycle and apoptosis related genes prior to activation (Figure 4) and display cell cycle defects and increased apoptosis after *in vitro* and *in vivo* activation. However, if the deletion of Ezh2 in naïve CD8^+^ T cells occurred only after activation in the Ezh2-g-KO mice, only cell cycle defect but no increase apoptosis could be observed in these activated naïve CD8^+^ T cells. This shows that cell cycle regulators (*Cdkns*) are more sensitive to the loss of Ezh2 than apoptosis related genes, and Ezh2 plays a critical role in repressing the expression of *Cdkn* genes during naïve CD8^+^ T cell activation-induced proliferation.

Expression of cell cycle inhibitors such as *Cdkns* is tightly regulated in T cells (9). Here we show that loss of Ezh2 resulted in altered expression of several key cyclin-dependent kinase inhibitors including *Cdkn2a* (p16 and Arf) and *Cdkn1c* (p57) in naïve CD8^+^ T cells after activation, which explained the cell cycle impairment of Ezh2-deficient naïve CD8^+^ T cells in response to antigenic stimulation. However, loss of Ezh2 had limited impact on other key *Cdkns* including *Cdkn2c* (*p18*), *Cdkn2d* (*p19*), *Cdkn1a* (*p21*), *Cdkn2b* (*p15*), and *Cdkn1b* (*p27*) (Figure S2 in Supplementary Material). In addition, a dramatic increase (70-fold) in expression of *Cdkn1c* (p57) in Ezh2 deficient naïve CD8^+^ T cells after 4 h stimulation also contributed to p53 mediated apoptosis pathway (8, 9) and increased apoptosis along with alteration of apoptosis-related genes. These findings provide evidence of the regulatory role of Ezh2 in cell cycle inhibitors and apoptosis related gene expression *via* modification of H3K27me3 levels at these loci. Loss of Ezh2 during thymocyte development resulted in apparent alteration of gene expression observed in freshly isolated and 16 h stimulated naïve CD8 T cells. But this transcriptional difference became less obvious at 72 h stimulation probably reflecting the selection of those less damaged dividing cells. However, in addition to the direct effects of Ezh2, the impairment of cell cycle progression of naïve CD8^+^ T cells over the course (several days) of analysis after stimulation in the absence of Ezh2 could also be secondary effects resulting from the defects of gene expression regulators controlled by Ezh2. Thus, it was necessary to dissect the direct and indirect effects of Ezh2 in regulation of cell cycle and apoptosis during naïve CD8^+^ T cell activation.

Applying GzmbCre-mediated Ezh2 deletion allowed us to examine the early effects of losing Ezh2 in naïve CD8^+^ T cells and differentiated cell cycle defects from apoptosis. Several reasons could explain the less severe defects observed in naïve CD8^+^ T cells from Ezh2-g-KO mice compared to naïve CD8^+^ T cells from Ezh2-c-KO mice. First, it takes time for the effect of Cre-mediated Ezh2 deletion to be observed phenotypically. Ezh2 deletion occurred in thymocyte development in Ezh2-c-KO mice whereas after activation of naïve CD8^+^ T cells of Ezh2-g-KO mice. The different degree of impairments reflects the degree of cumulated defects in the absence of Ezh2. Second, deletion of Ezh2 was completed in CD8^+^ T cells of Ezh2-c KO, whereas we observed various degree of deletion of Ezh2 in stimulated naïve CD8^+^ T cells from Ezh2-g-KO mice by single cell genotyping. Less than half (45%) of activated naïve CD8^+^ T cells had complete and one allele deletion of Ezh2, indicating that the impact of activation-induced immediate deletion of Ezh2 in naïve CD8^+^ T cells of Ezh2-g-KO mice could have been more severe if it were feasible to remove cells containing the undeleted Ezh2 loci from the analysis. Together, these findings show the sensitiveness of Ezh2 in processes involved inactivation-induced naïve CD8^+^ T cell proliferation.

The importance of cell division for T cell function during an infection is apparent yet the precise time required for the resting naïve CD8^+^ T cells completing first and subsequent cell cycle has not been directly measured. Previous methods calculates indirectly estimated the average cell division time based on the dilution of cell division tracking dye (32–34). In this study, we used live imaging to record cell divisions of naïve CD8^+^ T cells from WT, Ezh2-c-KO, and Ezh2-g-KO mice in response to *in vitro* stimulation with anti-CD3/CD28. To increase our efficiency of tracking motile T cells and their subsequent divisions over a long period of time, we cultured stimulated T cells in silicone inserts and imaged the entire chamber. The seeding number of cells was also optimized to accommodate their growth over the course of 72 h. With these optimizations in place, we directly observed several cells that underwent first and second round of divisions in response to stimulation. The average time for naïve CD8^+^ T cells to complete the first division was approximately 28 h. The deletion of Ezh2 in naïve CD8^+^ T cells resulted in significant delay of the first cell cycle completion. Although the subsequent second cell division took approximately one-third of time (8.5 h) in WT mice, the time of second cell division was further delayed in both Ezh2 KO mice. These observations reflect the cumulative defects in the absence of Ezh2. The live imaging setup can also track the localization and dynamics of specific fluorophore-tagged proteins and can serve as a crucial tool for understanding role of key regulators in cell division. In conclusion, our findings reveal a critical role of Ezh2 in regulation of cell cycle during naïve CD8^+^ T cell activation by establishing a repressing state of *Cdkn2a* and *Cdkn1c*.

## Ethics Statement

This study protocol was carried out in accordance with the guidelines of the National Institute on Aging and approved by the Institutional Review Board. Study subjects were participants of the Baltimore Longitudinal Study of Aging (BLSA) and gave written informed consent.

## Author Contributions

GC and N-pW designed experiments. GC, KS, SC, JL, and JK performed experiments. GC, SC, JL, and JK performed data analysis. AS helped array data analysis, XM and RW helped perform experiments. M-HS and N-pW supervised the research projects. GC and N-pW wrote the manuscript with approval from all authors.

## Conflict of Interest Statement

The authors declare that the research was conducted in the absence of any commercial or financial relationships that could be construed as a potential conflict of interest.

## Supplementary Material

Figure S1 Deletion of Ezh2 in Ezh2-c-KO CD8 T cells before and after *in vitro* stimulation. Naïve CD8^+^ T cells were isolated from spleen of WT or Ezh2-c-KO mice and stimulated with anti-CD3/CD28 *in vitro*. Cells were collected at 0, 24, and 96 h after stimulation and lysed in RIPA buffer. 10 mg lysate was separated on NuPAGE 4–12% Bis-Tris protein gel (Invitrogen). Protein was transferred to PVDF membrane and blocked with 5% milk in TBST buffer for 1 h. Membrane was then cut into two halves at 75 kDa based on molecular weight marker. Top part was probed with a-Ezh2 (#3147, Cell Signaling) at 1:2,000, bottom part was probe with a-actin (SC-47778, Santa Cruz) at 1:1,000, 4°C overnight. Membrane was washed with TBST and probed with HRP goat a-mouse (#405306, BioLegend) at 1:5,000, room temperature for 1 h. **(A)** Representative blot of Ezh2 and actin. **(B)** Relative level of Ezh2 was normalized to actin and the ratio of Ezh2/actin in WT cells at 0 h was set to one.

Figure S2 TUNNEL assay in Ezh2 deficient CD8^+^ T cells. WT or Ezh2-c-KO CD8^+^ naïve T cells were isolated and stimulated with anti-CD3 and anti-CD28 for 72 h *in vitro*. The cells were fixed with 1% (W/V) paraformaldehyde in PBS on ice for 15 min, and then resuspended in ice cold 70% ethanol, and incubated on ice for a minimum of 30 min. The fixed cells were labeled with BrdU with TdT enzyme for 60 min, and then stained with fluorescent conjugated anti-BrdU antibody. The cells were acquired on Flow Cytometer **(A)** and the percentages summarized in **(B)**. This figure is supplementary to Figure 2.

Figure S3 Gene expression profiles in Ezh2-deficient CD8^+^ T cells. Wild type or Ezh2-c-KO CD8^+^ naïve T cells were stimulated with anti-CD3 and anti-CD28 *in vitro*, and collected at different time points post-stimulation for qPCR analysis. The gene expression level was normalized to the level of Rpl32, and further normalized to the unstimulated wild type samples. This figure is supplementary to Figure 5.

Figure S4 Validation of Ezh2 deletion in naïve CD8^+^ T cells from Ezh2-g-KO mice. **(A,B)** WT or Ezh2-g-KO CD8^+^ naïve T cells were isolated, labeled with CellTrace Violet and stimulated with anti-CD3 and anti-CD28 *in vitro* for 4 days. **(A)** YFP expression was detected in the divided Ezh2-g-KO CD8^+^ T cells. **(B)** The YFP^+^ divided cells in Ezh2-g-KO and YFP- divided cells in WT were sorted with Flow Cytometry and used for Ezh2 mRNA expression by qPCR. The experiments were repeated twice. **(C–E)** Ezh2-g-KO genotyping. **(C)** Ezh2-g-KO or WT activated CD8^+^ T cells were sorted into undivided and >3 times divided population, and genomic DNA was isolated for Ezh2 genotyping by PCR. Lane L: DNA ladder. Lanes 1–6 were as following 1. WT, 2. Ezh2-c-KO, 3. equal mix of WT and Ezh2-c-KO, 4. undivided, and 5. >3 divided cells from Ezh2-g-KO and 6. water control. The up and low bands represented Ezh2^fl/fl^ and deleted Ezh2 PCR products, respectively. **(D)** Single Ezh2-g-KO or WT activated CD8^+^ T cells were sorted into 96 wells plate and genotyped for Ezh2 deletion by PCR. The representative gel image from one of the three repeats is shown. Lane L: DNA ladder. Lanes 1–16 were Ezh2-g-KO single cells, respectively. The up and low bands represented Ezh2^fl/fl^ and deleted Ezh2 PCR products, respectively. This figure is supplementary to Figures 6–8.

Figure S5 Tile scan profile of live cell imaging. WT, Ezh2-g-KO or Ezh2-c-KO CD8^+^ naïve T cells were labeled with CellTrace Far Red and stimulated with plate coated anti-CD3/CD28 (see Materials and Methods for details). The representative tile scan profile shows the complete imaging area of the chamber of interest in the silicone micro-insert. The edge effects as seen on the scan profile had no effect on data quantification. This figure is supplementary to Figure 8.

Video S1 Live Imaging of naive CD8^+^ T cells of WT mice. Time lapse movie (2 fps) of overlaid DIC and Far Red (CellTrace) channels of naive CD8^+^ T cells obtained from WT mice. A representative cell that was tracked over time is indicated with a white arrowhead. The cell undergoing first and second division is marked with cyan and yellow arrowhead, respectively. Duration in hh:mm at the top-left corner indicates the time of acquisition post-stimulation of the cells.

Video S2 Live Imaging of naïve CD8^+^ T cells of Ezh2-c-KO mice. Time lapse movie (2 fps) of overlaid DIC and Far Red (CellTrace) channels of naive CD8^+^ T cells obtained from Ezh2-g-KO mice. A representative cell that was tracked over time is indicated with a white arrowhead. The cell undergoing first and second division is marked with cyan and yellow arrowhead, respectively. Duration in hh:mm at the top-left corner indicates the time of acquisition post-stimulation of the cells.

Video S3 Live Imaging of naïve CD8^+^ T cells of Ezh2-g-KO mice. Time lapse movie (2 fps) of overlaid DIC and Far Red (CellTrace) channels of naive CD8^+^ T cells obtained from Ezh2-c-KO mice. A representative cell that was tracked over time is indicated with a white arrowhead. The cell undergoing first and second division is marked with cyan and yellow arrowhead, respectively. Duration in hh:mm at the top-left corner indicates the time of acquisition post-stimulation of the cells.
