# Supplementary figures and images for: Ezh2 Regulates Activation-Induced CD8+ T Cell Cycle Progression via Repressing Cdkn2a and Cdkn1c Expression
Source: Front Immunol. 2018 Mar 26;9:549. doi: 10.3389/fimmu.2018.00549 (PMC5879148; doi:10.3389/fimmu.2018.00549)

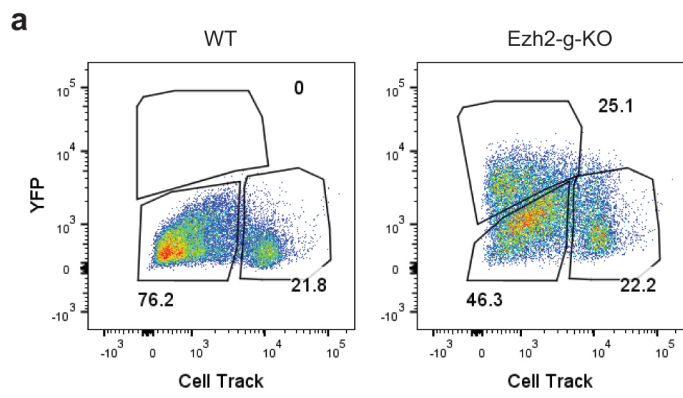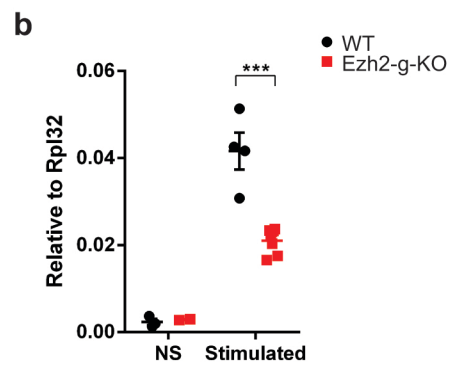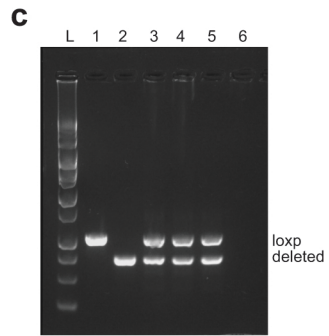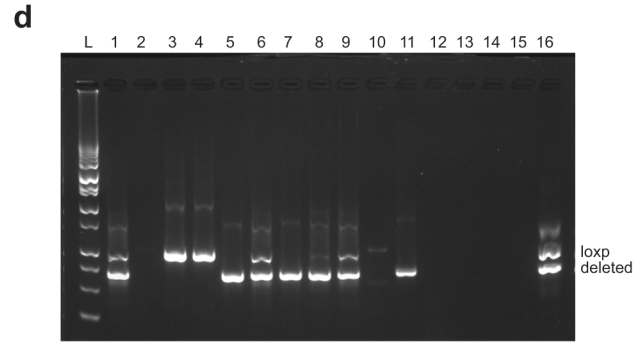

Supplement: Figure S4 — Validation of Ezh2 deletion in naïve CD8+ T cells from Ezh2-g-KO mice. (A,B) WT or Ezh2-g-KO CD8+ naïve T cells were isolated, labeled with CellTrace Violet and stimulated with anti-CD3 and anti-CD28 in vitro for 4 days. (A) YFP expression was detected in the divided Ezh2-g-KO CD8+ T cells. (B) The YFP+ divided cells in Ezh2-g-KO and YFP- divided cells in WT were sorted with Flow Cytometry and used for Ezh2 mRNA expression by qPCR. The experiments were repeated twice. (C–E) Ezh2-g-KO genotyping. (C) Ezh2-g-KO or WT activated CD8+ T cells were sorted into undivided and >3 times divided population, and genomic DNA was isolated for Ezh2 genotyping by PCR. Lane L: DNA ladder. Lanes 1–6 were as following 1. WT, 2. Ezh2-c-KO, 3. equal mix of WT and Ezh2-c-KO, 4. undivided, and 5. >3 divided cells from Ezh2-g-KO and 6. water control. The up and low bands represented Ezh2fl/fl and deleted Ezh2 PCR products, respectively. (D) Single Ezh2-g-KO or WT activated CD8+ T cells were sorted into 96 wells plate and genotyped for Ezh2 deletion by PCR. The representative gel image from one of the three repeats is shown. Lane L: DNA ladder. Lanes 1–16 were Ezh2-g-KO single cells, respectively. The up and low bands represented Ezh2fl/fl and deleted Ezh2 PCR products, respectively. This figure is supplementary to Figures 6–8. [file Image_4.PDF]

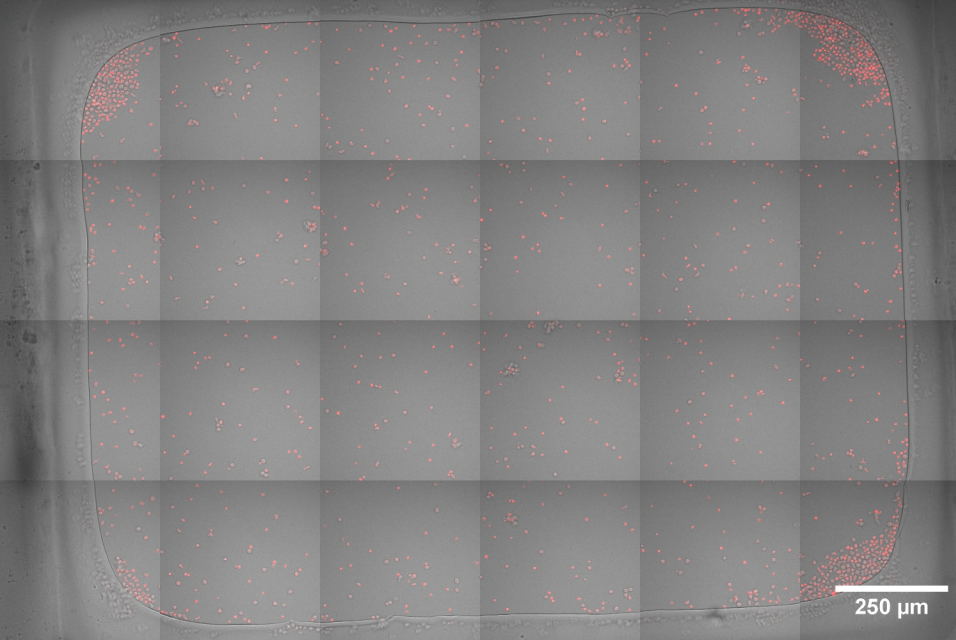

Supplement: Figure S5 — Tile scan profile of live cell imaging. WT, Ezh2-g-KO or Ezh2-c-KO CD8+ naïve T cells were labeled with CellTrace Far Red and stimulated with plate coated anti-CD3/CD28 (see Materials and Methods for details). The representative tile scan profile shows the complete imaging area of the chamber of interest in the silicone micro-insert. The edge effects as seen on the scan profile had no effect on data quantification. This figure is supplementary to Figure 8. [file Image_5.PDF]
